# HPV Vaccination Coverage Rate in a Rural Area: An Observational, Retrospective, and Cohort Study

**DOI:** 10.3390/vaccines10081274

**Published:** 2022-08-07

**Authors:** Lara Colomé-Ceballos, Josep Lluís Clua-Espuny, José Fernández-Sáez, Concepción Ceballos-García, Natàlia Andrés-Cubells, Maria Jesús Pla-Farnós

**Affiliations:** 1 Sexual and Reproductive Attention, SAP Terres de l’Ebre, Catalonian Health Institute, 43500 Tortosa, Spain; 2 Gynaecology Department, Hospital Verge de la Cinta de Tortosa, Catalonian Health Institute, 43500 Tortosa, Spain; 3 EAP Tortosa Est. Primary Care, SAP Terres de l’Ebre, Catalonian Health Institute, 43500 Tortosa, Spain; 4 Foundation Institute for Primary Health Care Research Jordi Gol i Gurina (IDIAPJGol), 43500 Tortosa, Spain; 5 Unitat de Suport a la Recerca Terres de l’Ebre, Fundació Institut Universitari per a la recerca a l’Atenció Primària de Salut Jordi Gol i Gurina (IDIAPJGol), 43500 Tortosa, Spain; 6 Unitat de Recerca, Gerència Territorial Terres de l’Ebre, Institut Català de la Salut, 43500 Tortosa, Spain; 7 Faculty of Nursing, Terres de l’Ebre Campus, Rovira i Virgili University, 43500 Tortosa, Spain; 8 Santa Creu de Jesús Hospital, Salut Tortosa, 43590 Tortosa, Spain; 9 Gynaecology Department, Hospital Universitari de Bellvitge, Carrer de la Feixa Llarga, s/n, 08907 L’Hospitalet de Llobregat, Spain

**Keywords:** vaccination, papillomavirus, coverage, equity, access, barriers

## Abstract

In order to reduce the incidence and mortality rate of cervical cancer, the World Health Organization (WHO) declared the Global Strategy Goal for 2030, advocating for reaching a vaccination coverage rate of >90% against human papillomavirus for girls by the age of 15 years. The main objectives of this study were (1) to determine the papillomavirus vaccination coverage among women 15–40 years old and (2) to identify the at-risk subgroups and possible barriers to achieving WHO’s 2030 goal. Multicentre, observational, retrospective, and community-based cohort studies were conducted on women from a rural area in southern Catalonia until 31 December 2021. A total of 23,136 women were included, with a mean age of 26.6 (SD = 5.6) years. The average dose number was 1.7 (SD = 0.7). The results showed overall vaccination coverage of 17.4% among the target women. This coverage was unequal across regions (16.6–24.5%, *p* < 0.001), primary healthcare teams (15.5–24.3%, *p* < 0.001), and age groups (56.7% (15–19-year-olds) vs. 3.8% (35–40-year-olds), *p* < 0.001), related to accessibility to vaccination and economic–geographical indicators. Clinical practice guidelines on screening individuals at risk in terms of vaccination access and public vaccination protocols should be implemented in order to improve the vaccination coverage rate.

## 1. Introduction

The association of human papillomavirus (HPV) with condylomatous, precancerous, and cancerous pathology [1,2] has been known for 40 years [3], and it is the most common sexually transmitted disease across the world [4]. While HPV infection is recognized as one of the leading causes of cancer, HPV is necessary but may not be sufficient for the development of cervical cancer [5,6] that is the second most common cancer affecting women aged 15–44 years. Currently, the vaccination against HPV and screening and treatment of pre-cancer lesions is a cost-effective way to prevent cervical cancer [7].

However, despite having effective screening technology [8] and HPV vaccines, there are significant differences between incidence and mortality by geographical region [9]. In the United States, the probability of developing HPV-related cancer is estimated to be between 1/362 and 1/158 over the life cycle [10]. In Europe, HPV causes approximately 44,000 cases of HPV-associated cancer [11,12]. Meanwhile, it is the most frequently diagnosed cancer in Africa [11,13]. On the contrary, in Spain, it is fourth in women aged 15–44 years, and the prevalence of known cervical infection is 13.6% [14]. Moreover, the incidence and mortality due to cervical cancer are significantly different across countries as a function of income [8,15] and social strata [16].

In order to tackle the progression of the incidence rates, the World Health Assembly proposed the Global Strategy Goal 2030 [8] to accelerate the elimination of cervical cancer, defining it as a public health problem that is sensitive to prevention and healthcare and strengthening immunization within primary health care. Existing computer systems in primary care centres can ensure the monitoring of population-based screening programs for cervical cancer and may be used to plan an organized vaccination program to ensure wider coverage and better follow-up [17] to obtain maximum coverage among the targeted population. Even with these tools and the availability of vaccination programs, there are no homogeneous data on the level of risk reduction following the application of the vaccine at an individual level [9,11,12] and the practice of population screening [18]. Although a reduction in HPV-associated pathology [19] has been estimated, the incidence rate prior to vaccine introduction is unknown [20], in addition to the prolonged period between vaccine application and observation of the impact on the population [20,21].

The territory of study is part of the phenomenon known as the “depopulation of European rural areas” [22]. It represents cytological coverage less than 20% compared to the rest of the regions, in the set of records of international cancer. The comparison of the incidence figures for this tumour places the Catalan registries in the lower part of the interval, and 80% of women with cervical cancer lack an adequate screening history. All this rather suggests a possible poor supply/demand screening and vaccination or a parallel use of screening systems’ non-homogenized information. Moreover, many public entities and scientific studies have established the distinctive elements of rural and urban spaces by relating the decline of the rural ones with factors such as aging, impoverishment, poor accessibility and coordination of health services [21], an unequal vaccination implementation HPV programs [22] by differences in the target population [23], type of vaccine used [22], and ineffective communication in certain population groups [19]. There is also experience in multiple health conditions that must be considered in order to improve screening programs [24,25] and vaccine hesitancy, which is well known to influence suboptimal vaccination coverage rates [26,27,28]. In this way, the in-depth analysis of the screening activity of the cervical cancer and coverage for HPV vaccination in Catalonia highlights the need to evaluate outcomes through both screening and prevention methods related to the HPV vaccine.

Therefore, the main objectives of this study were (1) to determine the papillomavirus vaccination coverage among women 15–40 years old and (2) to identify possible at-risk subgroups and barriers to achieving the WHO’s 2030 goal.

## 2. Materials and Methods

### 2.1. Study Design

This was a multicentre, observational, retrospective, and community-based cohort study conducted on women from a rural region in southern Catalonia from 1 January 2020 to 31 December 2021.

### 2.2. Study Scope

This study was conducted in the territory Terres de l’Ebre (Appendix A), which is divided into four regions—Baix Ebre (R1), Montsià (R2), Ribera d’Ebre (R3), and Terra Alta (R4)—and includes 191,791 inhabitants across 52 municipalities with an average of 54.5 inhabitants/km^2^ vs. 241.8 inhabitants/km^2^ in Catalonia [29]. The demographic evolution of Terres de l’Ebre involves an increase in the aging population (Table 1). It has the highest aging index (162.7) compared to Catalonia (127.1) and Spain (118.43) [30,31] as a result of low birth rates and a negative migratory balance. A loss of population is expected in the coming years, which will also contribute to the aging of the population. This is applicable in our study demographics, with the majority of the cohort involving the oldest population [32]. The average income per inhabitant is 77.4% vs. the Catalonia index of 100% [33].

The public health service is made up of four regions, with a total of 11 primary care teams (EAPs) and referring gynaecology services in each region, all of them are managed by the Catalan Health Institute, Department of Health (CatSalut). The reference hospital was the Hospital Verge de la Cinta in Tortosa, which is publicly managed through the Catalan Health Institute. The EAPs are organized as clinical functional units in terms of the administration and monitoring of the public vaccination schedule in schools. As a clinical reference, the Sexual and Reproductive Healthcare Center (ASSIR) provides partially decentralized services in the head centres of each primary care team through a midwife consultation, a gynaecology specialist team consultation dependent on primary care, and an obstetrics and gynaecology service as the main reference centre centralized in the Hospital Verge de la Cinta, where patients receive specialized care. The management of vaccination in people <15 years old is carried out by the paediatrics service, while vaccination in those ≥15 years old is carried out by the family nurse of the primary care team to which the patient is assigned.

Overall, 98.2% of the population registered in the population census from the territory has an active clinical history with at least one of the clinical centres of the territory. This digitalized clinical history availability allows continuous follow-up care from any centre (HC3).

### 2.3. Patients/Subjects of Study

Women aged 15–40 years old were included as participants in this study.

### 2.4. Observation Period

Data were available on the clinical history of the subjects until 31 December 2021.

### 2.5. Inclusion Criteria

The inclusion criteria were as follows:

- Active medical history at any of the clinical centres of the territory (HC3);
- Residents for ≥5 years in the study territory;
- Accessibility to registered variables and clinical data recorded.

### 2.6. Exclusion Criteria

The exclusion criteria were as follows:

- Patients without an active clinical history and/or not enough recorded data or inaccessibility to clinical data;
- Disease with a vital prognosis <1 year.

### 2.7. Variables

#### 2.7.1. Dependent Variable: HPV Vaccination Status

The primary outcome of our study was the vaccination coverage rate (VCR) in women aged 15–40 years old in Terres de l’Ebre. A patient was defined as properly vaccinated according to WHO position papers and CDC criteria HPV vaccination criteria: two-dose schedule for those who obtain the first dose before their 15th birthday; and a three-dose schedule for those who initiate vaccination at ages 15 through 45 years, and for immunocompromised persons [34,35]. The remainder of the population was considered to be either not properly vaccinated or not vaccinated.

There were three types of populations in our study according to vaccination cost: (a) G1, patients <15 years old who received their doses through public vaccination programs and, thus, had their doses publicly funded; (b) G2, patients ≥15 years old with approved risk criteria who had their vaccination publicly funded; (c) G3, patients who were vaccinated following medical counselling, which was not publicly funded.

Women in the first group (G1) were included in the vaccination program [36] of the Catalonia government, whose doses were publicly funded. Regarding the case of adolescents over 13 years old, only girls who were not previously vaccinated or who were partially vaccinated are to be vaccinated. Two doses are administered at least five to six months apart (depending on the vaccine used). If vaccination is started after 14 or 15 years of age, three doses are administered with a schedule of zero, one to two, and six months (depending on the vaccine used). Patients who meet the vaccination criteria according to the school calendar are vaccinated in schools by the paediatric service of the primary care team of the corresponding EAP and are registered in the clinical record. A vaccination schedule was considered complete when at least two doses of the vaccine were administered to those ≤15 years old.

Patients in the second group (G2) had risk criteria as defined by the Health Department of the Government [37], with access to financed vaccination. The following risk situations are considered: immunodeficiency (acquired or not), solid organ transplantation or hematopoietic precursors, use of immunosuppressive drugs, HIV infection (up to 26 years of age), men who have sex with men (up to 26 years of age), people in a situation of prostitution (up to 26 years of age), and women who have had cervical surgery (any age). Patients who meet the risk factor criteria are vaccinated by the primary care team of the corresponding EAP and are registered in the clinical record. In order to access the vaccine financially, a professional must detect that the patient meets the criteria and then make the request to the vaccination centre. The vaccine arrives at the EAP and is administered by the nurse in charge. In our study, a vaccination schedule was considered complete when at least two doses of the vaccine were administered [36,37,38].

The final group included women not in the previous groups (G3) without access to publicly funded doses, but who paid for the vaccination. There were also patients who did not meet the criteria for the school vaccination schedule or risk factors, but received vaccination following advice from a healthcare professional. These patients purchased the vaccine at their pharmacy with a prescription from the advising professional, and it was administered by their nurse. In our study, a vaccination schedule was considered complete when at least two doses of the vaccine were administered [36,37,38]. The remaining women not included in these groups were considered “incompletely or not vaccinated”.

The vaccination coverage rate was calculated by region, primary healthcare team, age group and vaccination cost group.

There are three vaccines currently available worldwide. In this area, and in all cases where public funding is possible, the vaccine administered is 9vHPV, with two doses in the population <15 years old. However, from 2008 to 2017, the vaccine administered was 4vHPV (from 2008 to 2014 with three doses, and from 2014 to 2017 with two doses) [36]. In the case of people not meeting the criteria for funded doses, the professional decides the HPV vaccine to be administered.

#### 2.7.2. Independent Variables

The independent variables were as follows:

(1) Sociodemographic variables: Age, region of residence, assigned primary care team (EAP), aging index (people ≥65 years old per 100 people ≤15 years old), and annual per capita average income as the relative percentage of each region vs. Catalonia (100%).
(2) Clinical variables: Number of administered doses, type of vaccine, date of administration, HPV vaccination (G1, public vaccination program funded for target population; G2, funded vaccination according to risk criteria; G3, nonfunded vaccination), cervical screening test and cytology-registered, HPV polymerase chain reaction test (PCR)-registered, and HPV-positive PCR diagnosis. These variables were described by vaccination status. All presented results have been stratified by age.

### 2.8. Data Collection and Information Sources

All participants were managed by the Catalonian Health Institute (ICS) through 11 primary healthcare teams (EAPs). The clinical background data were obtained retrospectively in an anonymized fashion from a computerized database, provided by the Information and Communication Technology Department from the minimum basic dataset at hospital discharge (CMBD-HA) register using the specific International Classification of Diseases (10th version; ICD-10) to the principal investigator in a fully deidentified format. The data were supervised and analyzed according to the General Data Protection Regulation of Spain/Europe from 1 February 2017. The study was conducted in accordance with the most relevant standards regarding data handling, concerning the experimental context with patients, ethics, and data protection and privacy, following Directive 95/46/EC (protection of individuals regarding the processing of personal data and on the free movement of such data). All the data were included in an ad hoc repository, which was delivered to the main researcher. The study protocol received the ethics evaluation and approval from the Ethical Committee of Jordi Gol University Institute of Primary Care Research with a registration number 21/064-P.

The datasets utilized for this project were as follows:

1. The 11 primary care teams all managed by the Catalonian Health Institute (Governmental agency) shared clinical information database for all general practice (E-cap, HC3) and hospital (E-sap) interactions, including clinical data, symptoms, investigations, diagnoses, comorbidities, prescribed medication, referrals to secondary and tertiary care, and status (alive/death) of 97.7% of resident people as of 31 December 2021. Pharmacological variables were collected from the SIRE (Catalan acronym for Integrated Electronic Prescription System).
2. The HC3 Shared Clinical Record in Catalonia (CatSalut, Health Department), i.e., the Patient Episode Dataset for Catalonia, which includes demographic and clinical data on all inpatient and outpatient daily admissions in Catalonian hospitals.
3. The Institute of Statistics of Catalonia for each region of the territory, including percentage gross disposable household income/inhabitant vs. the Catalonian average (100%), inhabitant density/km^2^, and aging index vs. Catalonia (100%) [19,31,34]. Data on these factors were collected automatically when possible, or manually otherwise.

### 2.9. Statistical Analysis

The vaccination coverage rate was calculated by dividing the number of women with all doses of the complete vaccine by the total target population.

The data are presented using frequencies and percentages for categorical variables and using means with standard deviations for continuous variables. A descriptive analysis of the variables according to region and Catalonia was carried out using frequencies and percentages for the categorical variables and the means and standard deviations for the continuous variables.

To detect differences between the two groups, we used the χ^2^ test for categorical variables and the Mann–Whitney U-test for continuous variables. Performed contrasts were two-sided. The statistical package IBM SPSS Statistics 20 was used for all analyses. Statistical significance was set at *p* < 0.05.

## 3. Results

### 3.1. Vaccination Coverage Rate Obtained in the Target Population

#### 3.1.1. Description of the Sample

A total of 23,136 women were included, covering 85.9–100% of the census target population (N = 24,415). They represented 26.3% of all women and 13.0% of the total population in Terres de l’Ebre (Appendix A). The mean age of the sample was 26.6 (SD = 5.6) years, distributed as follows: 15–19-year-olds (17.4%), 20–24-year-olds (16.8%), 25–29-year-olds (16.9%), and 30–34-year-olds (19.7%). The 35–40-year-old group was significantly more populated (29.1%; *p* < 0.001). Table 1 describes the basal characteristic of the study population.

#### 3.1.2. Vaccination Coverage and Screening Test Results

The HPV vaccination program had coverage of 17.4% among the target population. A total of 7388 (31.6%) women were registered as having initiated papillomavirus vaccination (PVV), whereby they received at least one dose of any HPV vaccine. A total of 6360 patients (74.8%) were completely vaccinated according to the public health system program in G1, 353 patients (8.7%) were completely vaccinated according to risk criteria in G2, and 675 patients (16.4%) were completely vaccinated by their own means following medical council in G3. The mean age of the vaccinated people was significantly lower than that of the unvaccinated people (21.0 (SD = 5.4) years vs. 31.9 (SD = 5.9) years; *p* < 0.001) (Table 2).

The percentage of people ≤15 years old completely vaccinated by the public schedule was 49.9% (48.8–52.2%) without significant differences among regions, but there were significant differences when the overall target population was considered by region (16.6–24.5%; *p* < 0.001), primary healthcare team (15.5−24.3%; *p* < 0.001), and age group (56.7% for 15–19-year-olds vs. 3.8% for 35–40-year-olds; *p* < 0.001). The average dose number was 1.7 (SD = 0.7). Overall, 81.8% of vaccinated people were administered the 4vHPV vaccine (*p* < 0.001). The papillomavirus vaccination coverage showed significant differences. According to the study, unvaccinated women (37.1%) were more likely (*p* < 0.001) to undergo cervical cancer screenings than those who were vaccinated (10.7%), and they had more frequent HPV screening PCR (*p* < 0.001) but lower HPV-positive test prevalence (*p* < 0.001) (Table 2).

Reviewing complete VCR results stratified by age and according to vaccine group, a VCR of 56.4% was observed in 15-19 years, 17.6% in 20–24 years, 4.7% in 25–29 years, 0.0% 30–34 years, and 35–40 years in G1 (globally 26.6%); and 0.2% was observed in 15–19 years, 0.8% in 20–24 years, 7.1% in 25–29 years, 6.4% in 30–34 years and 3.8% in 35–40 years in G2+G3 vaccination groups (globally 7.7%), respectively (Table 3).

Regarding cervical cancer screening stratified by age, greater screening in older patients was observed and distributed as follows: Cervical cytology globally 28.6%, with a mean age 33.3 years old (SD = 4.8): 0.2% in 15–19 years old, 6.5% in 20–24 years old, 32–40% in 25–29 years old, 44.0% in 30–35 years old and 45.8% in 35–40 years old; HPV screening PCR test globally 4.7%, with a mean age 34.2 (SD = 4.6): 0.0% in 15–19 years old, 0.9% in 20–24 years old, 3.9% in 25–29 years old, 7.1% in 30–35 years old and 8.7% in 35–40 years old; and Positive Result HPV screening PCR test increased progressively from 18.9% (35–40 year-old) up to 58.8% (20–24 year-old) with a total prevalence of 20.4%. 10.6% of the PCR tests performed on unvaccinated women were positive vs. 50.5% of the tests performed on vaccinated women.

### 3.2. Identification of Risk Groups

Region 4 had the highest VCR (24.5%; *p* < 0.001), while Region 1 had the lowest (16.6%; *p* < 0.001). (Appendix A). The 35–40-year-old cohort was the group with the lowest VCR (3.8%; *p* < 0.001). According to economic–geographical indicators, the highest vaccination coverage rate was obtained in the region with the highest aging index, the lowest percentage of the target HPV population, and the lowest population density of inhabitants per square kilometre.

Regarding incomplete vaccination (Table 4), among those <15 years old, 50.0% had received a single dose of the vaccine, without significant differences according to region, 36.5% (2322 patients) had received two doses, and 13.4% (852 patients) had received three doses. In contrast, among those ≥15 years old (1028 patients; 13.9% of those vaccinated), 71.9% had received a complete vaccination schedule, while 28.0% had an incomplete schedule (150 patients—14.5% had received a single dose; 138 patients—13.4% had received two doses).

By type of vaccine administered, the most administered vaccine in both categories was 4vHPV. Among those who were vaccinated under 15 years of age, 84.2% (5357) were vaccinated with 4vHPV, 9.7% were vaccinated with 2vHPV, and 6.0% were vaccinated with 9vHPV. Among those >15 years of age, 4vHPV was also predominant (63.4%), followed by 9vHPV (27.9%) and 2vHPV (8.6%).

### 3.3. Barriers to Vaccine Accessibility

The oldest participants (35–40 years old) were those with the lowest VCR (3.8%). The lowest global VCR (16.6%) was obtained in the most populated region, which was closest to the gynaecologist reference centre. The least populated region (Region 4) was the region farthest from the reference centre. This region had the highest global VCR (24.5%), but the lowest VCR in G1 (60.3% VCR in G1 in Region 4 compared to 74.8% VCR in G1 in all regions). Data on the male population were unavailable.

## 4. Discussion

We described a population of 15–40-year-old patients with a complete vaccination coverage rate for HPV of 17.4%. This low coverage rate is associated with age group, because 49.9% of the target population (<15 years old, vaccinated according to the criteria of the public vaccination schedule) had received a complete vaccination with at least two doses of any HPV vaccine, representing the highest vaccination coverage obtained in the region with the highest aging index, the lowest percentage of the target HPV population, and the lowest population density of inhabitants per square kilometre.

The international data available on vaccination coverage in adult women are very limited and refer to selected populations and age groups or include women in catch-up programs, making comparisons difficult. Although vaccination rates have been rising since the first HPV vaccination programs, only 59% of 13–17-year-olds were fully vaccinated in 2020 [39]. Around the world, the results show an average coverage of 67% for vaccination initiation and 53% for the complete schedule. In Europe, the vaccine coverage in terms of last dose achieved ranges from 35% (France) to 97% (Malta). In Spain, a vaccine coverage of 84% in terms of the last dose has been reported [40] in women included in vaccination programs, but the cumulative vaccination coverage against HPV in women aged 15–55 years not included in a current vaccination program remains very low and is slowly rising [39]. In Catalonia, vaccine coverage of 73.5% in terms of the last dose has been reported [40].

Herein, unequal and lower results were observed in terms of vaccination coverage, both at the individual region level and at the national or regional level. We found a lack of uniformity in the implementation of criteria for the vaccination protocol. Regarding G1, we observed a VCR of 56.7% in patients 15–19 years old and a VCR of 18.5% in patients 20–24 years old. The increase in vaccine coverage observed for G1 at the age of 20–24 years, ranging from 18.5% to 56.7% with respect to coverage at the age of 15–19 years of age, is a reflection of publications in the national media regarding possible side effects [41].

Given significant differences between age groups in HPV positivity, this group is especially important because this HPV prevalence curve has already been observed (highest among young women aged 20–24 years, with a constant decline with age) and the VCR of the G1 group is the main predictor of the future HPV protection and related comorbidities among the adult population.

However, coverage of HPV vaccination, coverage of cervical screening, and the HPV positivity rate are age dependent but different according to age group and accessibility group (G1 vs. G2 vs. G3). Factors such as participation, cytological classification system, country, continent, collection method, analysis limited to the prevalence of the four types of HPV that are vaccine-preventable, and year of publication may have a statistically significant impact. The combination of lower cervical cancer screening rates and low HPV vaccine uptake represents a critical challenge in cervical cancer prevention. This raises the need to assess a change in protocol in public vaccination in order to protect these women, including the detection of high-risk individuals in the Health Promotion and Preventive Activities Protocol as a systematic goal in primary care (PAPPs) to be closer to the WHO’s goal of a VCR of ≥90%. The availability of a cervical cancer screening program, the detection of high-risk individuals, and monitoring according to homogeneous and shared quality criteria [42] could guarantee the implementation of measures that bring us closer to the objectives proposed by the WHO. Targeting any group is an option to consider ensuring equity of access and to improve the effectiveness of the HPV vaccination program. Countries in all income groups must devise strategies to achieve and maintain higher levels of HPV immunization [40].

Given a total prevalence of 20.4%, a significantly higher percentage of HPV–PCR positivity among vaccinated (50.5%) vs. non-vaccinated (10–6%) women may seem paradoxical. It could be explained in part by the average age of each population (31.9 year old (SD = 5.9) for non-vaccinated vs. 21.0 year-old (SD = 5.4) for vaccinated, and 32.4 year old (SD = 5.2) for HPV-positive) or by risky sexual behaviours. We must note the possible selection bias in patients selected to perform HPV PCR determination, as it depends on the criteria of the professional or the screening of risk behaviours. If we compare the results with those available in the literature, we can find a prevalence of 13.6% in the Canary Islands [14], 19.0% in China [43], and 43.8–55.8% in Kazakhstan [44]. It could be interpreted as a registry bias, a bias in clinical practice and/or an inherent result associated with the characteristics of this population.

Some barriers can be identified. The vaccination schedule is different across territories in Spain, depending on the autonomous communities, because each department of health determines the type of vaccine, as well as when and how it is administered. However, in the case of the HPV vaccine and the school vaccination schedule, there is a consensus at the national level, and two separate doses are administered five to six months apart to 12-year-old girls [42]. Even the management of the school vaccination program by the EAPs according to differences in population and territorial extension may be related to the different vaccination rates obtained.

Given that HPV vaccination initiation and completion are strongly associated with the use of health services [45], the characteristics of the study territory could include other possible barriers. The phenomenon of rural–urban migration and rural depopulation in Europe and the rural–urban dichotomy have established the distinctive elements of rural and urban spaces by relating the decline of the rural ones with factors such as aging, poor accessibility to health services, or poverty, among others. The importance of this research lies in the fact that Spanish rural areas are located and characterized, which have worrying data in terms of demography, economy, health service accessibility on the already limited healthcare facilities available in rural areas. This territory has a scattered and very low-density population, with long travel times to referral services, which may condition access to vaccination and specialized care required by patients with risk factors for contracting HPV. From 2015 to 2018, the percentage of parents who declined the HPV vaccine for their kids due to safety concerns nearly doubled; however, beyond the public concern about HPV vaccine safety [46], the advice of health professionals regarding vaccines, from nurses, family doctors, and referring specialists, has been defined [47] as one of the more effective interventions to improve HPV vaccination coverage [48,49,50]. There is restricted access to funded vaccination; only patients who meet the risk factor requirements (G2 in our study), patients who fit into the school calendar (G1), and girls belonging to G1 but are not vaccinated due to inaccessibility have the catch-up option in our protocol. Differences by age group are attributed to access to the school vaccination protocol. Women over 26 years of age were not vaccinated by the program but belong to the group with risk factors for later developing cervical cancer.

It could be that patients fulfilling the risk criteria did not have access to vaccination due to various circumstances, e.g., a health professional not identifying the risk factors and indicating the vaccination, being unable to request public-funded vaccination, or not having access to private vaccination due to current cost. All these data are similar to the results from a European multicentre study [19] showing a lack of funding for vaccination, a lack of information on the benefits of HPV vaccination, and the lack of proactive vaccination recommendations by health professionals as the principal factors that most condition the acceptability of vaccination.

Overall, 10 out of 27 countries in the EU currently have or have committed to having their vaccination protocols inclusive for boys, this meaning a change in public health politics towards gender equality [23]. The guidance on human papillomavirus (HPV) vaccination in EU countries [47,51,52] considering a female-only HPV vaccination of pre-adolescent girls is probably more cost-effective at the current vaccine cost, but it does not sufficiently protect men who have sex with men. In fact, the routine recommendation for HPV vaccination in adolescents is not conditional on sexual activity in any country; it is less equitable and less resilient to sudden drops in vaccine uptake. Data are not available in our population on VCR regarding men, but it raises ethical doubts about achieving the goal sought if half the population does not have access to vaccination, which should be considered, on the basis of available evidence and risk criteria, whether men ought to be included in the vaccination program and/or HPV vaccination should be included as an option for not only primary prevention but also secondary prevention.

These results suggest that HPV vaccination and cervical cancer screening rates remain low among the target population of young women. Interestingly, this study warns about groups with different rates of HPV vaccination and brings up various conditions in the territory to improve the coverage of HPV vaccination, as listed below:

1. Significant differences in vaccination coverage between neighbouring regions;
2. Women aged ≥25 years old (excluded by age from funded vaccination) do not have subsequent differentiated screening or opportunistic monitoring for HPV clinical guidance in primary care;
3. Guidance of clinical practice toward the systematic screening of high-risk candidates for HPV vaccination in primary care;
4. Avoidable conditions restricting access to funded HPV vaccination (e.g., age, income, temporal residence, or low accessibility to gynaecologist professionals);
5. Monitoring public policies according to homogeneous criteria about the vaccination coverage achieved and sharing of corrective decisions;
6. Men’s coverage, especially high-risk candidates for HPV vaccination as primary or secondary prevention.

Lastly, the COVID-19 pandemic has affected health systems worldwide. Restrictions related to population mobility, as well as the collapse and closure of health centres, could have had a major impact on vaccination HPV coverage that is largely unknown.

The importance of this research lies in the fact that Catalan rural areas are located and characterized, which have worrying data in terms of demography, economy, and health services accessibility on the already limited healthcare facilities available in rural areas [19]. It also had some limitations, such as the non-inclusion of male population data because currently they are excluded from the HPV Catalan vaccination program. The results are obtained from the records of the study population and could have led to a registry bias, a bias in clinical practice and/or an inherent result associated with the characteristics of this population. The authors used all analyses on the use of registered cohorts, an approach that circumvents the comparability limitations posed by the heterogeneous data available between countries and subnationally within countries, and it should be considered an implicit and common limitation associated with the registration system and territorial organization. The study design does not allow for answering this limitation. Eventually, the results will offer a baseline for measuring the early impact of available vaccines and for monitoring changes in HPV prevalence and the HPV-tested rate. Likewise, the possible conclusions of this work can provide and improve clinical alternatives to similar regions.

Ongoing studies [53,54,55] will provide evidence on certain identified research gaps concerning HPV vaccination and allow for additions and updates to this guidance to assess the independent prognostic factors for increasing the vaccination coverage, especially among the at-risk subgroups, as well as on the existence of preventive resources accessible to the target population excluded from the HPV vaccination. As possible future research, it is proposed to use tools [24,25] in order to identify opportunities for behaviour changes against the proposed screening and/or vaccination programs such as those described in this study.

## 5. Conclusions

We described a population of 15–40-year-old patients with a complete vaccination coverage rate for HPV of 17.4%.

The VCR showed significant differences across regions (16.6–24.5%), primary healthcare teams (15.5–24.3%), and age groups (56.7% for 15–19-year-olds vs. 3.8% for 35–40-year-old).

The highest VCR was obtained in the region with the highest aging index, the lowest percentage of the target HPV population, and the lowest population density per square kilometre.

This study warns about groups with different rates of HPV vaccination and brings up various conditions in the territory to improve the VCR.

The use of clinical practice guidelines for systematic/opportunistic screening of individuals at risk in terms of advice and vaccination access protocols should be implemented. Data on the male population are not available.

## Figures and Tables

**Table 1:** Basal characteristics by region (Terres de l’Ebre, 2020).

	Region 1		Region 2		Region 3		Region 4		All Regions	
Age Group	*N*	(VCR%) ^1^	*N*	(VCR%) ^1^	*N*	(VCR%) ^1^	*N*	(VCR%) ^1^	*N*	(VCR%) ^1^
15–19	1756	55.5	1613	57.5	449	58.6	241	56.9	4059	56.7
20–24	1765	17.6	1484	18.6	462	20.2	196	22.4	3907	18.5
25–29	1728	9.8	1494	12.3	484	11.9	224	24.5	3930	11.8
30–34	2015	6.2	1773	6.1	515	3.9	241	16.6	4544	6.4
35–40	3020	4.2	2678	3.4	678	2.5	320	7.5	6696	3.8
All	10,284	16.6	9042	17.5	2588	17.4	1222	24.5	23,136	17.4
**Average age (years (SD)) ^2^**										
G1 ^3^	19.2 (2.9)		19.7 (2.9)		20.3 (5.3)		19.7 (3.3)		19.2 (2.9)	
G2 ^4^	30.4 (6.8)		29.9 (6.4)		29.7 (5.6)		27.5 (7.5)		29.4 (6.6)	
G3 ^5^	28.6 (7.6)		28.6 (7.7)		28.3 (7.5)		28.2 (7.6)		28.4 (7.6)	
Non-vaccinated	31.8 (6.0)		32.1 (5.9)		31.6 (5.9)		32.1 (5.8)		31.9 (5.9)	
Vaccinated	21.0 (5.4)		20.7 (5.2)		20.6 (4.7)		22.1 (5.9)		21.0 (5.4)	
All	26.4 (5.7)		26.7 (5.6)		26.1 (5.3)		27.1 (5.9)		26.6 (5.6)	
Aging index	153.5		151.4		200.7		247.9		162.7	
Average income/inhabitant	79.1		73		84.3		79.3		77.4	
Inhabitant density/km^2^	78.5		93		26.4		15.3		54.5	

^1^ VCR, complete vaccination coverage rate; ^2^ SD, standard deviation; ^3^ G1, <15 years old, properly vaccinated by funded doses; ^4^ G2, ≥15 years old with risk criteria, properly vaccinated by funded doses; ^5^ G3, properly vaccinated without funded doses.

**Table 2:** Percentage vaccination, cervical screening test, and HPV PCR by region.

	Region 1	Region 2	Region 3	Region 4	All Regions
	Complete Vaccination (%) According to Group				
G1 ^1^	75.3	75.8	78.9	60.3	74.8
G2 ^2^	9.2	8.7	4.6	11.0	8.7
G3 ^3^	15.4	15.4	16.4	28.6	16.4
	**Cervical screening test and cytology (%) according to vaccine provider registration**				
**Average age**					
(years (SD) ^4^)	33.4 (4.7)	33.3 (4.9)	33.2 (4.5)	33.5 (4.4)	33.4 (4.7)
Not vaccinated	42.8	31.1	32.5	44.4	37.1
Vaccinated	11.9	9.4	3.5	13.6	10.1 ^6^
All	33.3	24.2	23.4	31.6	28.5
	**HPV screening PCR ^5^ (%) according to vaccine provider registration**				
**Average age**					
(years (SD) ^4^)	34.2 (4.5)	34.5 (4.7)	31.8 (5.4)	35.3 (4.1)	34.6 (4.4)
Not vaccinated	6.7	4	2.8	6.5	5.2
Vaccinated	4.7	3	1.7	3.3	3.6 ^6^
All	6.1	3.7	2.5	5.2	4.7
	**HPV-positive PCR ^5^ (%) according to vaccine provider registration**				
**Average age**					
(years (SD) ^4^)	32.5 (4.8)	33.3 (5.2)	27.2 (4.3)	34.6 (5.4)	32.4 (5.2)
Not vaccinated	0.6	0.4	0.5	1.1	0.5
Vaccinated	2	1.9	1.3	0.9	1.8 ^6^
All	1	0.9	0.8	0.9	0.9

^1^ G1, <15 years old, properly vaccinated by funded doses; ^2^ G2, ≥15 years old with risk criteria, properly vaccinated by funded doses; ^3^ G3, properly vaccinated without funded doses; ^4^ SD, standard deviation; ^5^ PCR, polymerase chain reaction; ^6^ *p*-value between vaccinated and non-vaccinated population (*p* < 0.001).

**Table 3:** Complete HPV Vaccination, Screening Tests, and Results by Age group.

Age Groups	15–19	20–24	25–29	30–34	35–40	Total
**Complete HPV Vaccination/Total Vaccinable Group ^1^**						
**G1**	2295	692	187	0	0	3174
	(56.4%)	(17.6%)	(4.7%)	(0.0%)	(0.0%)	(26.6%)
**G2 + G3**	10	32	281	295	260	878
	(0.2%)	(0.8%)	(7.1%)	(6.4%)	(3.8%)	(7.7%)
**Cervical Screening Test and Cytology/Total for Age Group**						
**Average Age**						33.3 (4.8)
**(years, (SD))**						
**N**	7	253	1279	2008	3083	6630
**(%)**	(0.2)	(6.5)	(32.4)	(44.0)	(45.8)	(28.5)
**HPV Screening PCR Test/Total for Age Group**						
**Average Age**						34.2 (4.6)
**(years, (SD))**						
**N**	1	34	155	324	583	1097
**(%)**	(0.0)	(0.9)	(3.9)	(7.1)	(8.7)	(4.7)
**Positive Result HPV Screening PCR Test/Total HPV Screening PCR Test**						
**Average Age**						
**(years, (SD))**						32.4 (5.2)
**N**	0	20	47	65	92	224
**(%)**	(0.0)	(58.8)	(30.3)	(20.1)	(18.9)	(20.4)

^1^ Total number of patients meeting criteria for being included in a vaccination group.

**Table 4:** Number of doses administered by age group and region.

		Region 1		Region 2		Region 3		Region 4		All Regions	
Age Group	Number of Doses Administered	*N*	(%)	*N*	(%)	*N*	(%)	*N*	(%)	*N*	(%)
15–19	0	272	15.4	210	13	75	16.7	29	12	586	14.4
	1	508	28.9	476	29.5	111	24.7	75	31.1	1170	28.8
	≥2	976	55.5	927	57.4	263	58.5	137	56.8	2303	56.7
20–24	0	582	32.9	445	29.9	133	28.7	36	18.3	1196	30.6
	1	873	49.4	763	51.4	236	51	116	59.1	1988	50.8
	≥2	310	17.5	276	18.6	93	20.1	44	22.4	723	18.5
25–29	0	1523	88.1	1280	85.6	417	86.1	160	71.4	3380	86
	1	35	2	31.0	2	9	1.8	9	4	84	2.1
	≥2	170	9.8	183	12.2	58	11.9	55	24.5	466	11.8
30–34	0	1862	92.4	1650	93	494	95.9	198	82.1	4204	92.5
	1	29.0	1.4	14	0.7	1	0.1	3	1.2	47	1
	≥2	124	6.1	109	6.1	20	3.8	40	16.6	293	6.4
35–40	0	2873	95.1	2571	96	658	97	293	91.5	6395	95.5
	1	20	0.6	16	0.6	3	0.4	3	0.9	42	0.6
	≥2	127	4.2	91	3.4	17	2.5	24	7.5	259	3.8
All incompletely or not vaccinated		8577	83.4	7456	82.4	2137	82.5	922	75.4	19.092	82.5
All completely vaccinated		1707	16.6	1586	17.5	451	17.4	300	24.5	4044	17.4

**Figure A1:**
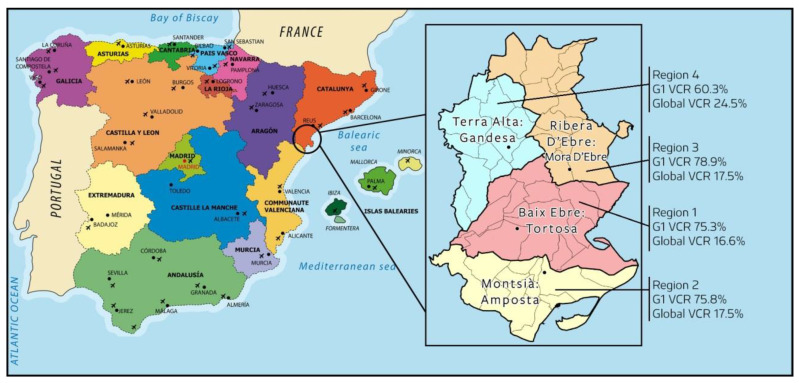
Global vaccination coverage rate by region.

## Data Availability

The data that support the findings of this study are available from the corresponding author (L.C.-C.) upon reasonable request.
